# Education Value Units: A Currency for Recognizing Faculty Effort

**DOI:** 10.5811/westjem.2022.11.57595

**Published:** 2022-12-28

**Authors:** Braden J. Hexom, Katarzyna Gore, Scott A. Heinrich, Yanina A. Purim-Shem-Tov

**Affiliations:** Rush University Medical Center, Department of Emergency Medicine, Chicago, Illinois

## BACKGROUND

Graduate medical education residency program funding is increasingly at risk where teaching institutions are faced with significant budgetary challenges.^1^,^2^ These funding challenges are compounded by recent revisions by the Accreditation Council for Graduate Medical Education (ACGME) redefining core faculty protected time to a 0.1 full time equivalent (FTE) benchmark.^3^ When based on 1,550 clinical hours per year, this represents a decrease in ACGME-required protected time by approximately 0.05 FTE per core faculty. It is imperative for programs to develop systems explicitly tracking time spent on educational activities.^4^ Some departments have developed education value units (EVU) to assign time and financial values to educational activities.^5^–^8^ Described as an initiative of the Association of American Medical Colleges to encourage institutions to approach fiscal management based on their missions, the concept of EVUs is borrowed from its clinical equivalent, the relative value unit.^9^

Our emergency department (ED), recognized a need to holistically quantify overall faculty effort sustaining our program and incorporate it into the department’s fiscal structure. Change management and workplace-based motivational theories, borrowed from the business literature, can inform principles for making such transitions.^10^–^11^ Challenging the status quo requires planning, structure, leadership, individual empowerment, communication, and frequent assessments highlighting wins. Furthermore, many individuals’ extrinsic motivators rest on achievement recognition and growth opportunities. Using these concepts to create new systems can lead to successful adaptation and acceptance from faculty stakeholders.

## OBJECTIVES

We developed an EVU system encompassing the educational and administrative responsibilities for our three-year emergency medicine (EM) residency program, of 36 total residents. Our objectives were as follows:

1. To quantify all activities necessary to run a high-quality residency program, including less often quantified activities such as mentorship, wellness, and faculty development;
2. To create a structure that acknowledged faculty effort, encouraged faculty to take ownership of their contributions, and hold them accountable for their activities;
3. To make an equitable distribution of tasks and compensated time in which efforts are rewarded and are reflected in appropriate time distribution.

We hoped to demonstrate to GME leadership that both core faculty and clinical faculty provided significant educational support to the residency program, and that this effort should be recognized with protected time to sustain those activities. Funding for GME at our institution is limited due to the number of residents exceeding the Medicare Direct and Indirect Payment limits (“GME cap”)^2^, and increasingly scarce institutional funding for faculty, especially with new ACGME faculty time definitions. However, our institution allowed for each department to internally fund a model to protect faculty time beyond the 0.1 FTE benchmark by redistributing unfunded protected time to all faculty. Previously, core faculty were defined as having 0.15 FTE specifically to support the residency mission. Clinical faculty did not have this time but may have received other FTE support (ie, from the medical school).

## CURRICULAR DESIGN

Our clinical faculty were given the opportunity to participate in up to 0.1 FTE of departmentally funded education time. We secured additional provisions from the institution to continue funding 10 core faculty at an additional 0.05 FTE for the pilot year (0.15 FTE total for core faculty). Next, we met with each faculty member to review their interests, skills, and past education efforts and asked them to consider their ideal effort level. We then assigned a level of effort each faculty would commit to in the following academic year. This ranged from 0 FTE to 0.1 FTE for some clinical faculty, to 0.15 FTE for core faculty, and 1.2 FTE total for three program leaders.

A faculty group consisting of departmental, residency, and fellowship leaders, as well as both core and clinical faculty, compiled a list of all hours required to sustain residency activities. This organizing group accounted for 24% of the faculty roster. The group tallied lectures, small groups, labs, professional development sessions, and administrative activities. A modified nominal group design was used across several meetings and emails where lists of hours were generated, shared, discussed, and revised. A final list curated by residency program leadership was presented at faculty meetings and further revised with core and clinical faculty input. Expanding from other EVU models, we chose to recognize diverse activities such as recruitment, mentorship, wellness, faculty development, rotation/track administration, and administrative tasks/activities we consider essential for a healthy residency program. Using the following assumptions, EVUs needed for the year were determined as follows:

- *Didactic Sessions:* Lectures; labs; board preparation; journal club; intern orientation (1,523 hours [hrs])
- *Lecture Preparation:* New lectures and old lectures with updates (296 hrs)
- *Conference Attendance:* Core faculty only (874 hrs)
- *Rotation and Elective Teaching*: Toxicology; addiction; ultrasound; anesthesia; and electives (1,290 hrs)
- *Recruitment*: Holistic application review, hosting webinars, residency fairs, interviewing applicants (1,399 hrs)
- *Program Administrative Time:* Evaluations; curricula development; data management; coaching; conference coordination; scheduling; performance reviews; crisis management meetings (1,693 hrs)
- *Program Standing Committees:* Participation in Clinical Competency Committee, Program Evaluation Committee; Diversity Equity and Inclusion Committee (482 hrs)
- *Faculty Development:* Workshops; retreats; planning (414 hrs)
- *Mentorship and Wellness*: Formal mentee relationships; career development; team building; retreats; community service; and mentoring Women in Emergency Medicine; Quality Improvement, and other groups (528 hrs)
- *Rotation, Elective, and Track Administration:* Non-teaching time for directing the simulation; ultrasound; toxicology; Foundations small-group series; pediatrics; addiction; social and global EM; a required Diversity Inclusion and Racial Equity program; and others (720 hrs)

Where discrepancies were identified, consensus among department, fellowship, and residency leadership led to an accepted EVU value. For instance, as described elsewhere, faculty’s self-reported lecture preparation ranged from 2–60 hours per lecture.^6^ The group agreed to categorize lectures as *de novo* (12 hrs credit) or updated (4 hrs credit). Similarly, EVUs were assigned to other didactics commensurate with the amount of preparation needed to create and/or deliver that content. Faculty could thus self-select the activities they would contribute (Table). Excluded were activities unrelated to resident education, such as medical student and fellow teaching. Although fellows are considered faculty in our program, their teaching is a requirement of their fellowship program. Also excluded were activities better described as personal/professional development such as publications, research, or teaching outside the EM residency.

The program was administered through a process of accountability and adjustment throughout the academic year. During the year, faculty proposed several new activities not initially included but that were deemed by residency leadership necessary for the training program (ie, an orthopedics curriculum). These activities were given credit, granted they sustained the training mission, thus allowing some faculty to adjust their contributions mid-year to meet their predetermined commitments. Accountability was achieved by reviewing the didactic calendar and various sign-up lists, and through individual communication, tabulating hours quarterly and distributing these as dashboards. Some end-products served as confirmation of task completion (ie, applicant screening, evaluations, committee reports). Faculty identified as behind in their contributions met with program leadership to consider alternative activities or revise their expectations with a change in their shift commitments.

## IMPACT/EFFECTIVENESS

The pilot was successful in quantifying a broad set of activities to describe the time required to administer a high-quality EM residency program. Faculty protected time was distributed to various degrees, with nine faculty taking 0.03–0.05 FTE (50–84 hrs); eight taking 0.08–0.1 FTE (135–168 hrs); nine taking 0.13–0.15 FTE (218–252 hrs); two associate program directors (APD) taking 0.35 FTE (588 hrs); one program director (PD) taking 0.5 FTE (840 hrs); and six taking no protected time. In total, 3.72 FTEs were distributed. The total number of hours predicted for education activities was 9,219, of which 3,412 were funded through the department (2.02 FTE) and 2,871 by the GME office (1.7 FTE - PD/APD/core faculty) for a total of 6,283 hrs (3.72 FTE). At the pilot conclusion, faculty had provided 8,416 hrs, or 2,133 hrs (1.26 FTE) more than were funded. These volunteer hours represent a significant effort by faculty to teach well above their level of funded support.

We underestimated by around 50% the time credited for lecture preparation. This was likely due to faculty seeing *de novo* lectures as “worth” more hours, and several wrote entirely new lectures (an outcome that program leadership felt was a positive influx of new content). Some clinical faculty who previously had not volunteered to teach found that they had an aptitude for it. Others who initially signed up for lectures switched instead to focus on small-group activities, mentorship, or administrative tasks. Similarly, mentorship and wellness hours were underestimated by around 15%, likely due to ongoing expansion of these programs. We overestimated by 20% the hours required for program leadership administrative time, although these were still well in excess of funded time. Similarly, we overestimated by more than 50% the hours committed to faculty development, and 30% to recruitment, likely due to mid-year redesigns of the program. All other categories were within 10% of the predicted number of hours.

Contributions of hours were tracked for 38 non-fellow faculty. This fulfilled our second objective of creating an accountability structure to acknowledge faculty effort. Three faculty left prior to completing a full year, although their activities were included in final totals. At the end of the year, 12 of 35 faculty were within 20 of their target hours. Faculty projected to not meet target hours were offered to increase their shift obligations the following year (one individual) or to take on additional tasks (four) including serving on the Clinical Competency Committee, managing a board review program, or expanding a telehealth curriculum. Several faculty members contributed hours well in excess of their protected time, including six who volunteered 50–100 additional hours, two faculty 100–150 additional hours (simulation and Foundations faculty), four faculty 200–250 additional hours (ultrasound and simulation faculty), and three program leaders 350–500 additional hours (Figure).

Our third objective to make an equitable distribution of tasks and compensated time was accomplished throughout the year with distribution of quarterly dashboards to faculty with subsequent adjustment of expectations. At end-of-year evaluations, department leadership used faculty members’ achievement of assigned activity goals as one marker for faculty success. Those well above their goals had some excess hours applied to the departmental incentive bonus formula. This pilot, with its focus on accountability and flexibility, has supported institutional requests to continue funding faculty time. We anticipated that some faculty would not meet the anticipated number of hours they had proposed. In fact, almost all faculty found activities to meet their projections, although with some mid-year adjustment.

As shown in Figure 1, faculty of all types provided more educational commitment than were funded. The implementation of this pilot has been influential in both the ED and institution. Faculty uptake has been favorable, and several have better defined their scholarly niche or charted a more deliberate promotion track. Our ED maintained core faculty time by demonstrating to GME that their contributions greatly exceed the modest hours reductions assigned to them. Several other departments are considering adopting this strategy.

Overall, this process describes a framework by which a currency of EVUs can be used to distribute effort, maintain the residency program mission, and provide accountability between leadership and faculty. This was a change management process that was collaborative and self-directed, with attention to a wide range of faculty interests, skills, and goals. Department leadership is more able to monitor faculty educational productivity yet allow for faculty to identify their ideal level of contribution and area of focus. It seems clear that the ACGME core-faculty benchmark of 0.1 FTE is not sufficient to recognize the contributions required to maintain a high-quality residency program.

Limitations of our design were most evident in the exclusion of non-ACGME fellows when quantifying faculty effort, despite the reality that they provided significant educational inputs. Related to this issue, the faculty who provided the most unfunded time, aside from program leadership, were fellowship directors and fellowship faculty. Our FTE distribution does not allow for any specific time to support non-ACGME simulation and ultrasound fellowship programs.

Future directions will use this framework in funding discussions with institutional leadership and to establish faculty support metrics to guide incentive funding. We anticipate that when periodically provided with individualized reports of contributions, faculty will have more agency to rebalance future clinical time with educational responsibilities. The innovation we describe will most likely be successful in academic centers where all faculty are assumed to have a commitment to medical education and desire to contribute to an educational mission. This is, therefore, unlikely to be replicated in programs where only a few core faculty provide most of the non-clinical teaching, although we speculate our framework could be modified to accommodate a variety of departmental FTE structures. We ultimately see this innovation as one tool to recognize all faculty who contribute to education in our ED in diverse ways tailored to individual skillset or personality and aligned with their professional and academic goals.

## Figures and Tables

**Figure:**
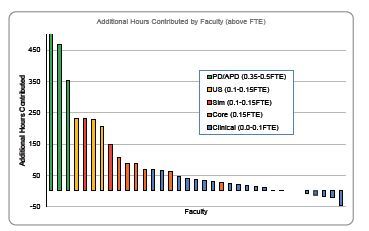
Additional hours contributed by faculty above the amount funded. *PD*, program director; *APD*, assistant program director; *US*, ultrasound; *Sim*, simulation; *FTE*, full time equivalents

**Table:** Selection of activities available to faculty and corresponding education value units (in hours offered per event/faculty).

Selected program activities (EVUs per faculty, number required)	Description of activity
Lectures (1 hr each, 65 total)	Classic educational talk
Lecture preparation (*de novo*) (12 hrs, 4 total)	Prep time allotted for creating new content
Lecture preparation (update) (4 hrs, 61 total)	Prep time allotted for updating preexisting talk
Foundations (case-based learning) (2 hrs, 32 sessions × 7 faculty)	Case-based small-group interactive learning
Small groups (5 hrs, 4 sessions × 3 faculty)	Small-group teaching on themed topics with multiple facilitators
Simulation labs (4 hrs, 20 sessions × 6 faculty)	Simulation case learning in small groups (usually 3–4 cases)
Procedure labs (3.5 hrs, 2 sessions × 8 faculty)	Hands-on, interactive simulation labs focused on procedures
5-minute radiology/ECG bundle (0.5 hrs, 32 sessions)	Interactive review of ECGs and radiology images as a large group
Ultrasound lab (4 hrs, 10 sessions × 3 faculty)	Hands-on, interactive ultrasound labs
Oral boards prep (2 hrs, 6 sessions)	Small-group review of oral boards style cases
SimTastic (10 hrs, 6 faculty)	Simulation competition with multiple stations
SonoGames (20 hrs, 3 faculty)	Ultrasound competition with multiple stations
Resident olympics (5 hrs, 10 faculty)	Multi-station medical knowledge and procedural competition
Diversity Inclusion, and Racial Equity (DIRE) Didactic Sessions (1.5 hrs, 9 sessions × 4 faculty)	Asynchronous and didactic curriculum developed to enhance knowledge and encourage discourse on topics related to diversity, inclusion, and racial equity
DIRE simulation lab (4 hrs, 4 faculty)	Simulation focused on diversity, inclusion, and racial equity topics
Orientation sessions prep and teaching (3 hrs, 25 sessions)	Didactic education during the PGY-1 month-long orientation block
Orientation lab sessions (3 hrs, 2 sessions × 8 faculty)	Lab sessions including cadaver and simulation labs for PGY-1 orientation month
Board prep workgroups (1 hr, 36 sessions)	Board review sessions reviewing commonly missed questions
Citywide oral boards day (3 hrs, 12 faculty)	Oral boards style mock examinations collaborating with neighboring Chicago programs
Citywide oral boards administration (12 hrs)	Preparation and coordination of mock oral boards sessions
Social and global health didactic sessions (1 hr, 14 sessions)	Didactic sessions outside of core curriculum on topics related to social and global health
Cadaver lab (2 hrs, 8 sessions × 2 faculty)	Procedure training on cadavers
Journal club (3 hrs, 6 sessions)	Group meeting to review and critique current literature
In-Person elective teaching (10hrs/week × 12 weeks)	Teaching by EM faculty outside of clinical shifts on in-person electives such as observation medicine, addiction, ultrasound, EMS, etc.
Virtual elective teaching (4hrs/week × 28 weeks)	Teaching of online-based elective such as documentation and billing
Applicant screening (25 hrs, 75 apps/faculty × 14 faculty)	Review and appraisal of medical student applicants for residency positions prior to interview offers
Regional recruitment events (3 hrs, 3 sessions)	Representing the residency at events focused on recruitment of a diverse cohort of residents
Rank list meeting (4 hrs, 14 faculty)	Meeting to review and determine the final rank status of medical student applicants for residency positions
Interview day interviews (4 hrs, 15 sessions × 5 faculty)	Conducting interviews with prospective applicants
Webinar recruitment series (1 hr, 8 sessions × 3 faculty)	Represent the residency at web-based recruitment event
Program Evaluation Committee (PEC) (3 hrs, 2 sessions × 8 faculty)	PEC meets to review resident perceptions and experiences in rotations as well as overall within the program and makes recommendations for changes.
PEC member data work (4 hrs, 8 faculty)	Prep work for meeting in which members review written evaluations of rotations in order to summarize for meeting
Clinical competency committee (CCC) (3 hrs, 2 sessions × 10 faculty)	CCC meets to review resident performance and provide overall assessments of residents and make recommendations of remediation and advancement
CCC member prep work (4 hrs, 2 sessions × 10 faculty)	Prep work for meeting in which members review written evaluation of the resident in order to summarize for meeting
CCC member data work (6 hrs, 2 sessions × 10 faculty)	Post work after meeting in which members create written assessment statements of residents for biannual meeting
Annual faculty development retreat (4 hrs, 10 faculty)	Yearly meeting on a specific faculty development topic
Annual faculty development retreat planning (10 hrs, 3 faculty)	Planning for the yearly faculty development session, including creation of didactics as well as event planning
Faculty development workshop planning (20 hrs, 4 sessions × 5 faculty)	Planning of quarterly faculty development sessions, which include creation of didactic content and coordination of speakers/panels
Faculty development workshop (2 hrs, 4 sessions × 10 faculty)	Quarterly faculty development didactic sessions on various topics including feedback, mentoring, autonomy, etc.
Resident retreat (16 hrs, 8 faculty)	Serve as faculty mentors during annual two-day retreat
Resident retreat planning (8 hrs, 4 faculty)	Planning, coordinating, and organizing yearly retreat
Wellness event planning (2 hrs, 12 sessions)	Planning of wellness-themed events
Faculty assigned resident mentor (12 hrs, 36 faculty)	Participating in a yearlong mentoring relationship with a resident with goals to meet for 1 hr, monthly
Resident research mentorship (4 hrs, 12 faculty)	Mentorship focused on resident scholarly activity.
Resident lecture mentoring/review (1 hr, 36 sessions)	Reviewing and providing feedback on didactics created by residents
Quality improvement group mentorship (12 hrs, 6 faculty)	Mentorship focused on quality improvement projects, working with residents to create meaningful change based on cases brought forth for quality review
Orthopedics curriculum development (20 hrs)	Development of an asynchronous curriculum on the topic of orthopedics
Women in EM mentorship (12 hrs)	Mentorship focused on topics relevant to female physicians

*EVU*, education value unit; *ECG*, electrocardiogram; *PGY*, postgraduate year; *EM*, emergency medicine.
